# Sex-specific differences in patients with differentiated thyroid carcinoma and their possible impact on survival

**DOI:** 10.1007/s00259-026-07845-y

**Published:** 2026-03-21

**Authors:** Marieke Heinrich, Elias Blickle, Tim Jedamzik, Patrick Kohnle, Alexander Kerscher, Frederik Anton Verburg, Andreas K. Buck, Kerstin Michalski

**Affiliations:** 1 Department of Nuclear Medicine, University Hospital Würzburg, Würzburg, Germany; 2 Comprehensive Cancer Center Erlangen EMN, University Hospital Erlangen, Erlangen, Germany; 3 Department of Radiology and Nuclear Medicine, Erasmus Medical Center, University Rotterdam, Rotterdam, Netherlands

**Keywords:** Differentiated thyroid carcinoma, Sex-specific, UICC-stage, Outcome prediction

## Abstract

**Purpose:**

This study aimed to analyze survival outcomes in patients with differentiated thyroid cancer (DTC) compared to the general population, with particular focus on sex distribution, UICC staging, and age-specific mortality patterns.

**Methods:**

A retrospective survival analysis was conducted on 3186 DTC patients with extended follow-up. Overall survival (OS) and relative survival (RS; observed survival in patients/expected survival in population) were compared to age- and sex-matched general population data, using Monte-Carlo simulation. Multivariate Cox regression analysis was performed to identify independent prognostic factors.

**Results:**

Female predominance was confirmed in younger age groups with balanced distribution in the 40- to < 55-year cohort. DTC patients demonstrated superior OS compared to the general population, with improving RS over time. Female patients showed better survival outcomes than males and exceeded general population survival, while male patients achieved comparable survival. UICC stage I showed significantly lower mortality than all other stages. Only stage IVb consistently demonstrated worse outcomes than the general population. All age groups showed equal to superior survival compared to the general population, with patients ≥ 55 years demonstrating significantly improved RS from 5 years onward. Multivariate analysis identified sex, age at diagnosis, and UICC stage as independent prognostic factors for death.

**Conclusion:**

This analysis confirms generally favorable prognosis for DTC patients, demonstrating superior survival compared to the general population, due to increased health consciousness but also reflects the highly effective therapeutic regimen. Only patients ≥ 55 years with distant metastases (UICC stage IVb) consistently showed worse survival than the general population, emphasizing the critical importance of preventing disease progression.

**Supplementary Information:**

The online version contains supplementary material available at 10.1007/s00259-026-07845-y.

## Introduction

Differentiated thyroid carcinoma (DTC) represents the most common endocrine malignancy, accounting for approximately 90% of all thyroid cancers and generally demonstrates favorable long-term prognosis compared to many other solid malignancies [1, 2]. The incidence of thyroid cancer has increased significantly over recent decades, partially attributed to improved diagnostics and increased detection of smaller tumors [3–5]. Despite this rising incidence, mortality rates have remained relatively stable, raising important questions about the long-term survival of DTC patients relative to individuals in the general population [4, 6]. Long-term population-based survival data are crucial for evaluating whether DTC patients experience survival outcomes comparable to, better than, or worse than the general population, and whether these patterns vary across specific subgroups in Germany. Factors such as patient sex, age at diagnosis, and disease stage according to the Union for International Cancer Control (UICC) classification are known to influence prognosis in DTC [7–9]. However, most conventional survival analyses compare outcomes solely within patient cohorts without accounting for expected mortality in matched general populations over extended time periods.

This study aims to analyze long-term survival outcomes in patients with differentiated thyroid carcinoma compared to the general population, with comprehensive stratification by key demographic and clinical variables. This is a follow-up study of the evaluation by Verburg et al. from 2013, with more patients, longer follow-up and updated UICC staging [10]. We seek to characterize temporal patterns of survival differences and identify specific demographic and clinical subgroups that may experience survival outcomes divergent from population-based expectations over time.

## Materials and methods

### Patient cohort

This study is based on adult patients with DTC, who were treated at the University Hospital Würzburg between 01.01.1965 and 30.09.2023 and were registered in the Comprehensive Cancer Center Mainfranken (CCC-MF). The local Ethics Committee waived the need for further approval due to the retrospective character of the study (waiver no. 20231103 02).

The Onkostar system (Version 2.13.1–60, 2023, IT-Choice Software AG) was used for comprehensive cancer documentation, including diagnosis, therapy, and follow-up data through the CCC-MF. Additionally, all information from Onkostar was cross-checked with the electronic medical record, to ensure data quality. Due to the mandatory reporting of cancer cases to the responsible cancer registry, reliable recording of deaths is generally assured.

To ensure a uniform stage classification, all tumor stages were updated according to the 8th edition of the American Joint Committee on Cancer (AJCC) and the UICC classification, which came into effect on January 1, 2018. Key changes include raising the age threshold for stages I and II from < 45 to < 55 years and the elimination of stage IVc [7, 11]. Notably, the recently published 9th edition did not introduce modifications to the staging system for differentiated thyroid carcinoma; therefore, our findings are applicable to the current classification system [12].

The mortality rates of the study cohort were compared with those of the German general population using period life tables from the Federal Statistical Office. We considered the overall mortality and made no differentiation between cancer-specific and non-cancer-related deaths.

Overall survival was defined as the duration from the date of initial diagnosis to the date of death. For patients who were alive at the time of analysis, survival was censored at the date of last available follow-up, indicating their minimum observed survival time.

### Statistical analysis

The life expectancy of reference individuals was calculated using a Monte Carlo simulation. The period life tables published by the Federal Statistical Office were utilized, spanning the years 1900 to 2020 and matching was performed for sex and age at diagnosis, ensuring the reference population reflected the demographic structure of the study subjects. For each patient, the simulation of an entire life course was performed iteratively 1000 times, enabling a robust estimation of life expectancy [13, 14]. In each simulated year, survival or death was determined by a binomial random draw, based on the age- and sex-specific mortality probability [15]. The calculated life expectancy for each reference had to be at least equal to the patient’s age at diagnosis, in order to minimize sample bias. Median survival with 95% confidence interval (CI), was reported. Relative survival (RS) as (observed survival in patients)/(expected survival in matched general population), was estimated with the Ederer II method [16–18]. Overall survival was analyzed with Kaplan-Meier-curves, log-rank test and uni- and multivariable Cox-Regression. Differences were analyzed using Fisher’s exact test, chi-squared test, and t-test or Mann-Witney U Test. Data are presented as mean or median with 95% confidence interval (CI), Interquartile range (IQR) or range. The significance level was set at *p* < 0.05. The software R-Studio (R 4.4.1, MacOS Version 2023.09.1 + 494, 2023, Posit Software, PBC) and GraphPad Prism version 10.6.0 for Windows, GraphPad Software, Boston, Massachusetts USA were used for statistical analysis.

## Results

### Patient characteristics

Between 1965 and 2023, 3401 patients with DTC were treated at the University Hospital Würzburg. After excluding 215 cases due to missing information, 3186 patients remained, including 2240 women and 946 men. Median follow-up was 6.18 years (IQR 2.01–13.97) and did not differ between sexes (females 6.11 years; males 6.39 years; U = 1032904; *p* = 0.26). Median age at diagnosis was 48.39 years (IQR 36.70–59.85). Papillary carcinoma (PTC) accounted for 78% and follicular carcinoma (FTC) for 22% of cases. Most patients were diagnosed at UICC stage I (76%, *n* = 2405). All patients were treated according to the respective current German clinical guideline. See Table 1 for detailed information.

**Table 1:** Patient characteristics

Number [*n*] (%)	Overall	female	male
	3186 (100)	2240 (70)	946 (30)
Median Follow up [years] (IQR)	6.18 (2.00–13.97)	6.11 (1.83–14.36)	6.39 (2.31–13.29)
Median age at diagnosis [years] (IQR)	48.39 (36.70–59.85)	47.52 (35.68–59.41)	49.96 (39.28–60.82)
Age at diagnosis [n] (% of respective group)			
< 30	433 (14)	321 (14)	112 (12)
30 - <40	556 (17)	420 (19)	136 (14)
40 - <55	1081 (34)	749 (33)	332 (35)
55 - <70	800 (25)	539 (24)	261 (28)
70 - <80	271 (9)	174 (8)	97 (10)
> 80	45 (1)	37 (2)	8 (1)
Histology [n] (%)			
Papillary	2492 (78)	1802 (80)	690 (73)
follicular	694 (22)	438 (20)	256 (27)
UICC-stage 8th edition [n] (%)			
Stage I	2405 (75)	1726 (77)	679 (72)
Stage II	355 (11)	218 (10)	137 (14)
Stage III	32 (1)	22 (1)	10 (1)
Stage IVa	10 (1)	7 (< 1)	3 (1)
Stage IVb	164 (5)	98 (4)	66 (7)
Stage unknown	220 (7)	169 (8)	51 (5)

### DTC patients in comparison to the general population

The DTC patient cohort showed a median survival after age of diagnosis of 32.62 years (95% CI 29.82–36.13 years). The simulated age and sex matched population control showed a median survival after age of diagnosis of 26.00 years (95% KI 25.98–26.08 years). DTC patients had significantly improved survival compared with age and sex matched general population controls (HR = 0.82; 95% CI 0.75–0.89; *p* < 0.01), with relative survival significantly exceeding population levels from 5 years onward and remaining stable over 10-, 15- and 20-year follow-up (Supplement Table 1).

### Sex distribution comparison

Female patients were diagnosed at a slightly younger age than males (female: median 47.52 years vs. male: median 49.96 years; U = 977511; *p* < 0.01), so sex distribution varied by age group in Fisher’s exact test: women predominated below 40 years (30 - < 40-years: Relative Risk (RR) 1.30; 95% CI 1.09–1.56 for females; *p* < 0.01), distribution was equal between 40 and 55 years (RR 0.95; 95% CI 0.86–1.06 for females; *p* = 0.39), and men predominated between 55 and 80 years (70 - < 80-years: RR 1.33; 1.05–1.68 for males; *p* = 0.02). Women had a higher relative risk of PTC (RR 1.10; 95% CI 1.06–1.15; *p* < 0.01). UICC stage I was more common in women (RR 1.07; 95% CI 1.03–1.13; *p* < 0.01) and stages II and IVb more common in men (II: RR 1.49; 95% CI 1.22–1.82; *p* < 0.01 and IVb: RR 1.60; 95% CI 1.18–2.15; *p* < 0.01).

### Comparison of male and female patients with the general population

**Fig. 1:**
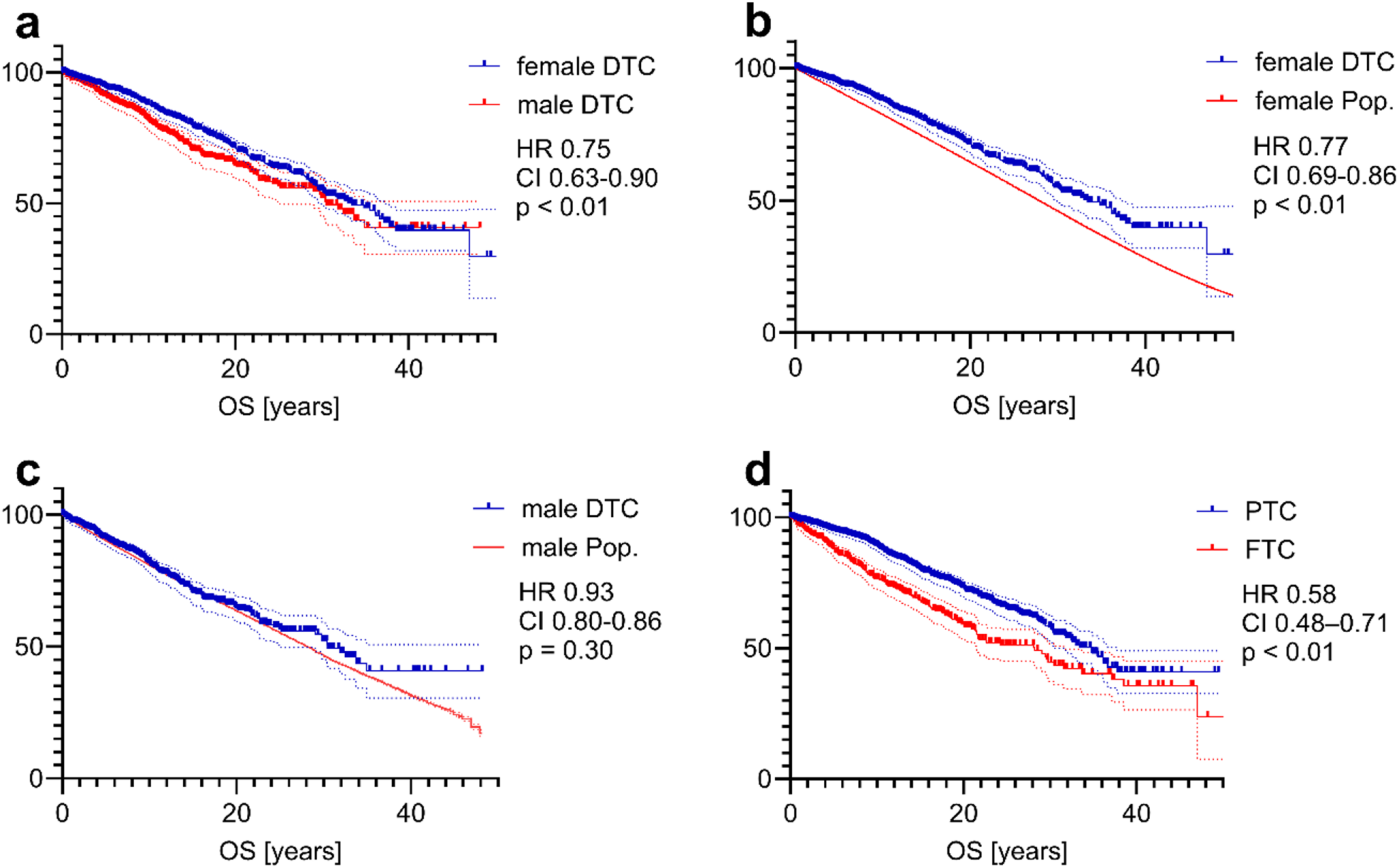
Female DTC patients showed significant improved OS compared to male DTC patients (**a**). Female DTC patients showed a significant improved OS compared to age matched female general population control (**b**). Male DTC patients showed comparable OS to age matched male general population control (**c**). PTC showed improved OS compared to FTC (**d**)

Abbreviations: CI: confidence interval; DTC: differentiated thyroid cancer; FTC: follicular thyroid cancer; HR: hazard ratio; OS: overall survival; Pop.: matched general population; PTC: papillary thyroid cancer, OS: overall survival.

Female DTC patients showed significantly longer median survival than male patients (33.42 vs. 30.45 years; Hazard Ratio (HR) 0.75; 95% CI 0.63–0.90; *p* < 0.01; Fig. 1a). Female DTC patients had a clear survival advantage compared to age matched female general population controls (median survival after age of diagnosis 33.42 vs. 27.84 years; HR 0.77; 95% CI 0.69–0.86; *p* < 0.01; Fig. 1b), with significant relative survival improvement from year 5 onward (Supplement Table 1). Male patients showed a comparable overall survival to age matched male general population controls (median survival after age of diagnosis 30.45 versus 26.14 years; HR 0.93; 95% CI 0.80–1.07; *p* = 0.30; Fig. 1c) and relative survival did not differ significantly from population levels at any time point (Supplement Table 1).

### Comparison of papillary and follicular DTC with the general population

PTC showed significantly longer median OS than FTC (PTC 34.86 vs. FTC 28.21 years; HR 0.58; 95% CI 0.48–0.71; *p* < 0.01; Fig. 1d). PTC patients also lived significantly longer than their age and sex matched general population controls, with relative survival improving continuously over time. FTC patients demonstrated survival comparable to the age and sex matched population, with relative survival clustering around 1 across all time points (Supplement Table 1).

### UICC stage comparison with the general population

**Fig. 2:**
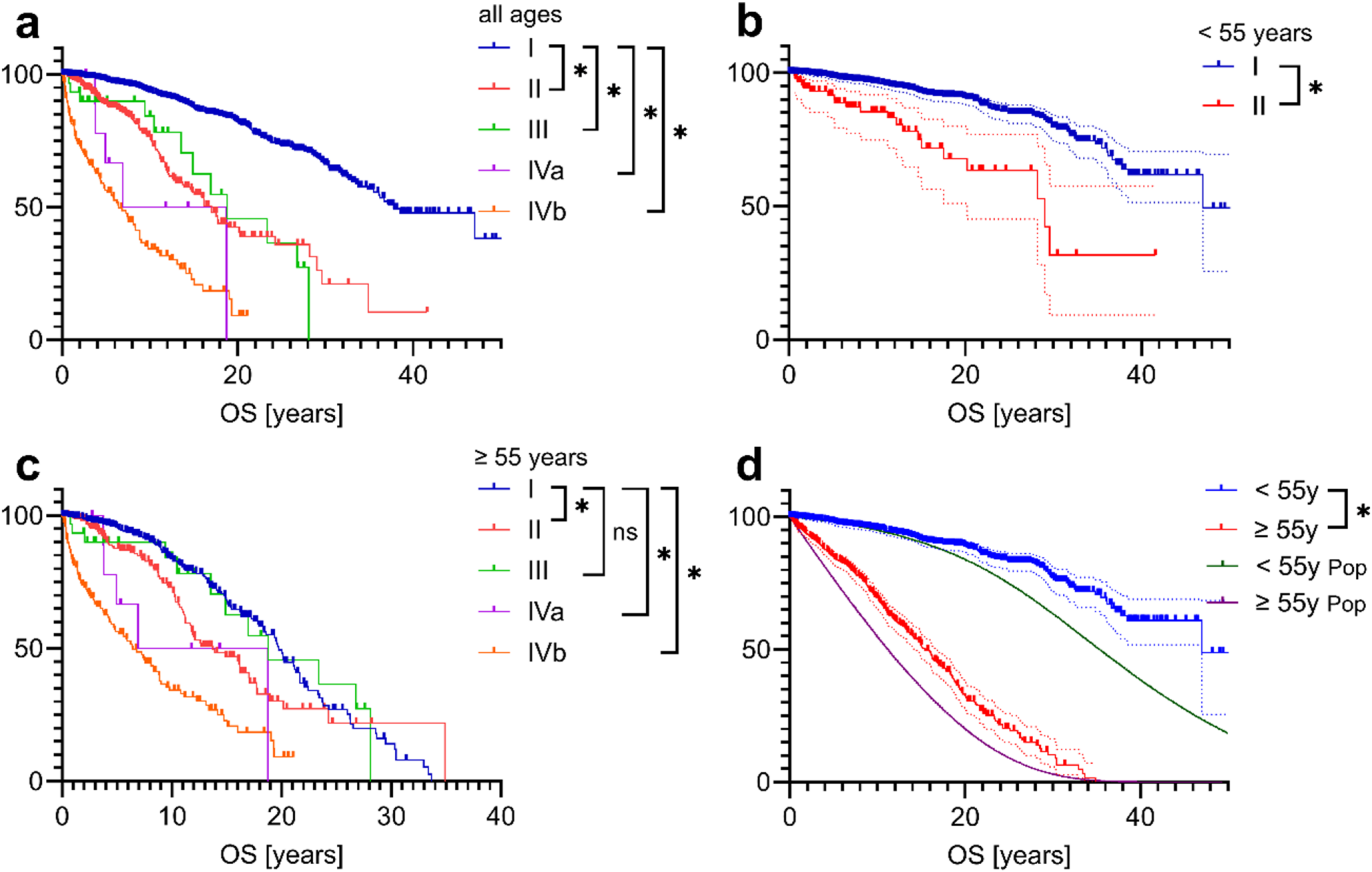
Across all age groups, higher UICC stage (I-IVb) was strongly associated with increased mortality in comparison to UICC stage I (**a**). In patients < 55 years, stage I showed a significantly better OS than stage II (**b**). In patients ≥ 55 years, mortality increased steadily with stage. However, stages I and III did not differ significantly (**c**). Patients < 55 years showed improved OS compared to patients ≥ 55 years (d). Patients < 55 years and patients ≥ 55 years lived significantly longer than the age and sex matched population (**d**)

Abbreviations: ns: not significant; OS: overall survival; Pop: matched general population; UICC: Union for International Cancer Control; y: years.

Across all age groups, higher UICC stage was strongly associated with increased mortality (Fig. 2a). Stage II, a heterogeneous group including younger patients with distant metastases and older patient with lymph node metastases only, showed a more than fourfold increased mortality risk compared with stage I (median survival 17.04 vs. 37.77 years; HR 4.18, 95% CI 3.28–5.30, *p* < 0.01). Stage III showed a similar elevation (median survival 18.79 vs. 37.77 years; HR 3.99, 95% CI 2.11–6.83, *p* < 0.01). Mortality increased progressively with stage, though stages II and III did not differ significantly in survival (log rank test *p* = 0.96; Fig. 2a). Age-stratified analysis showed in patients younger than 55 years a significantly better survival in stage I than stage II (median survival 47.0 vs. 29.0 years; HR 4.18; 95% CI 2.55–6.53; *p* < 0.01; Fig. 2b). In patients aged 55 years or older, mortality increased steadily with stage. However, stages I and III did not differ significantly (log rank test *p* = 0.84; Fig. 2c). Stage III patients received with median 6.68 GBq ^131^I a significantly higher cumulative activity than stage I patients with median 3.42 GBq ^131^I (U = 4376; *p* < 0.01).

Compared with the age and sex matched general population, stage I patients had markedly better survival, with relative survival advantages increasing continuously toward 20 years. Stage II patients had mortality comparable to their age and sex matched general population, with relative survival slightly elevated early but returning toward 1 later. Stage III patients demonstrated significantly lower mortality than the matched population and showed increasingly improved relative survival at later time points. Stage IVa did not differ significantly from the age and sex matched population cohort, aside from a small early relative survival advantage that likely reflects small sample size. Stage IVb patients showed significantly increased mortality and significantly reduced relative survival at 1, 5 and 10 years; later estimates were limited by diminishing sample size. Patients with unknown stage showed no significant difference from the reference population (Supplement Table 2).

### Age-Specific mortality compared to the general population

Patients younger than 55 years and patients starting with 55 years lived significantly longer than the matched population (< 55 years: median 47.01 vs. 35.35 years after diagnosis; HR 0.65; 95% CI 0.57–0.74; *p* < 0.01; ≥ 55 years: median 15.42 vs. 11.19 years after diagnosis; HR 0.65; 95% CI 0.60–0.70; *p* < 0.01; Fig. 2d). Survival patterns differed strongly by age at diagnosis. Patients diagnosed before age 30 had no detectable survival difference compared with the age and sex matched population, and relative survival remained consistently around 1 (Supplement Table 3). Those aged 30 to < 40 years showed small but significant relative survival advantages emerging at 10, 15 and 20 years. Patients aged 40 to < 55 years showed small but not significant RS advantages, with relative survival diverging significantly from the matched population only after 20 years. Patients aged 55 to < 70 and 70 to < 80 years showed improved survival compared with matched population controls, with relative survival increasing progressively from 5 years onward. Patients diagnosed at ≥ 80 years also showed significantly better overall survival than age and sex matched population controls, though limited follow-up led to absence of relative survival estimates beyond 10 years.

### Independent prognostic factors

Multivariate Cox regression showed that age at diagnosis, UICC stage, and sex were all independent prognostic factors for mortality. Each additional year of age increased mortality risk by 9% (HR 1.09, 95% CI 1.08–1.10; *p* < 0.01). Each increment in UICC stage increased mortality risk by 34% (HR 1.34, 95% CI 1.25–1.44; *p* < 0.0.1;). Male sex was associated with a 52% higher mortality risk (HR 1.52; 95% CI 1.25–1.84; *p* < 0.01), while female sex reduced mortality risk by 34% (HR 0.66; 95% CI 0.54–0.80; *p* < 0.01). The model showed strong discriminative performance (Harrell’s C = 0.83). Although age and UICC stage showed moderate multicollinearity, all variance inflation factors were within acceptable limits, indicating stable coefficient validity.

## Discussion

### Gender distribution comparison

The observed gender distribution confirms well-established epidemiological patterns, with a female predominance in younger age groups and a more balanced distribution in older cohorts, particularly in the 40 to 55-year age group. These findings align with Le Clair et al.´s observation that gender disparity is primarily confined to the detection of small subclinical PTC, which are identified more frequently in women during life despite equal prevalence at autopsy in both sexes [19].

Consistent with previous reports, female patients demonstrated superior survival outcomes compared to males, like Ries et al. showed before [20]. Our analysis revealed that women achieved better RS than the female matched population, while men showed comparable survival to the male population. This observation partially contradicts Verburg et. al´s findings of no marked difference in RS between genders [10]. The multivariate Cox regression analysis identified male sex as an independent negative prognostic factor for death, supporting the general observation by Radkiewicz et al. that men have higher cancer risk and worse prognosis [21] and confirming findings by Wilhelm et al. for male sex as an independent risk factor for increased risk of thyroid cancer death [22]. However, this contradicts Nilubol et al.´s assertion that sex is not an independent prognostic factor for disease-specific survival, but Nilubol et al. analyzed exclusively FTC [23].

### Comparison of papillary and follicular DTC with the general population

PTC demonstrated better overall survival than the age and sex matched general population, with RS improving over time, while FTC showed comparable survival to the general population. The comparison between PTC and FTC confirmed the established superior prognosis of PTC [24, 25].

### UICC stage comparison with the general population

Survival analysis demonstrated clear risk stratification according to UICC staging. A particularly notable finding was the nearly identical hazard ratios for UICC stages I and III in patients ≥ 55 years. This observation warrants careful consideration, as it suggests potential limitations in the discriminatory capacity of the current 8th edition of UICC staging system for these specific stages, despite Tuttle et al. and several other reports of improved survival curve separation in the 8th edition [7, 26, 27]. In contrast, the analysis in the preceding study from our study center showed better survival curve separation than in the current analysis when using the 7th edition UICC staging classification, in which stages II, III and IVa were additionally differentiated based on no lymph node metastases (UICC II), lymph node metastases in the central compartment (UICC III) and in the lateral compartment (UICC IVa) [10]. In our analysis, stage III patients received a significantly higher cumulative activity of ^131^I, so these findings might reflect the high effectiveness of an intensified therapy regiment in advanced stages.

Relative survival analysis revealed that UICC stages I II and III respectively demonstrated better overall survival compared to the age and sex adjusted population, as reported in the prior study from our university hospital [10]. Stage IVa showed no significant difference from the general population. Stage IVb was the only category demonstrating consistently worse outcomes than the general population throughout the observation period, confirming findings of Verburg et al. of clearly reduced life expectancy in patients with distant metastases [10].

### Age-Specific mortality compared to the general population

All age groups demonstrated superior overall survival compared to the general population. Patients under 30 years showed RS comparable to the general population, confirming findings of the prior study from our study center of normal life expectancy in patients younger than 45 years [10]. The 30 to < 55-year age group showed a trend toward improved RS beginning at 10 years, becoming significant at 20 years of follow-up. Patients aged 55 years and older demonstrated significantly improved relative survival beginning at 5 years, with progressive improvement over time.

These patterns may be explained by increased health consciousness and participation in screening programs, particularly those initiated at age 50. The significant improvement in RS at 20 years in the 30 to < 55-year cohort (when patients reach 50–75 years of age) and the early improvement observed in those aged 55 years and older support this hypothesis. Interestingly, these findings contrast with reports of the previous study from our study center of moderate life expectancy reduction in patients aged 45–59 and strongly reduced survival in those aged 60 and older [10], potentially reflecting changes in health awareness and healthcare delivery since 2013, but could also be a selective survivor effect. As the OS analysis is registry-based, we rule out observation bias favoring more health-conscious patients.

### Independent prognostic factors.

Multivariate Cox regression analysis confirmed sex, age at diagnosis, and UICC stage as independent prognostic factors for death, consistent with findings by Tuttle et al., Radkiewicz et al. and several others [7, 21]. In the absence of disease-specific survival analysis, age at diagnosis as a risk factor most likely reflects the natural increased mortality of older individuals rather than thyroid cancer-specific effects, especially since no age group demonstrated inferior relative survival compared to the matched population. With respect to sex, female sex should be interpreted as a protective factor rather than male sex as detrimental, given that male patients showed survival comparable to matched controls while female patients exhibited superior survival. Consequently, UICC stage represents the most relevant independent prognostic factor in our analysis.

### Limitations

This study’s limitations include the absence of a uniform treatment protocol due to its retrospective design. The extended observation period encompasses substantial evolution in therapeutic approaches and management strategies for DTC, making it challenging to attribute survival outcomes to specific interventions. However, the extended follow-up period constitutes a key strength, facilitating comprehensive overall survival analysis in DTC. As a single-institution registry from a high- volume specialized center, the external validity of our findings regarding treatment options may be limited in general treatment facilities and lower-volume centers. The retrospective nature may introduce potential selection bias inherent in registry-based analyses. No data regarding comorbidities and socioeconomic status were obtained. Although the matched design (age and sex) and the generally uniform access to healthcare in Germany make major imbalances in treatment access unlikely, comorbidities and socioeconomic factors remain confounders that could result in overestimation of survival. Furthermore, limited data for certain subgroups, particularly UICC stage IVa, warrant cautious interpretation of findings for these categories.

### Clinical implications

The superior survival outcomes observed across most patient subgroups compared to the matched population likely reflect multiple factors, including the indolent nature of most DTCs, effective treatment modalities, close medical surveillance, and potentially higher health awareness in DTC patients. The finding that only UICC stage IVb patients demonstrated consistently worse survival than the general population emphasizes the importance of preventing disease progression to distant metastasis and the need for improved therapeutic strategies for advanced disease, such as early high-dose radioiodine therapy after initial ablative radioiodine therapy.

## Conclusion

DTC can be effectively treated and is associated with improved survival in women and PTC compared to the matched general population, and to comparable survival in men and FTC. Only for patients > 55 years with distant metastases survival was reduced in comparison to matched population. The similar survival outcomes between UICC stages I and III in patients ≥ 55 years, suggests potential refinement opportunities in current staging systems to better discriminate prognosis in these stage groups.

## Supplementary Information

Below is the link to the electronic supplementary material.

Supplementary Material 1

## Data Availability

The datasets generated during and analysed during the current study are available from the corresponding author on reasonable request.
