# Supplementary material for: Sex-specific differences in patients with differentiated thyroid carcinoma and their possible impact on survival
Source: Eur J Nucl Med Mol Imaging. 2026 Mar 21;53(8):5042–9. doi: 10.1007/s00259-026-07845-y (PMC13249767; doi:10.1007/s00259-026-07845-y)
Supplement: Supplementary file 1 — Supplementary Material 1 [file 259_2026_7845_MOESM1_ESM.docx]

# Sex-specific differences in patients with differentiated thyroid carcinoma and their possible impact on survival

Marieke Heinrich^1^, Elias Blickle^1^, Tim Jedamzik^1^, Patrick Kohnle^1^, Alexander Kerscher^2^, Frederik Anton Verburg^3^, Andreas K. Buck^1^, Kerstin Michalski^1^

1 Department of Nuclear Medicine, University Hospital Würzburg, Würzburg, Germany

2 Comprehensive Cancer Center Erlangen EMN, University Hospital Erlangen, Erlangen, Germany

3 Department of Radiology and Nuclear Medicine, Erasmus Medical Center, University Rotterdam, Rotterdam, Netherlands

Corresponding Author: Dr. med. Marieke Heinrich; E-mail: heinrich_m4@ukw.de

**Supplement Table 1** DTC patients in comparison to matched general population

| Relative Survival | | Number [n] | RS | 95% CI | P value |
| --- | --- | --- | --- | --- | --- |
| DTC | 1 year | 2654 | 1.00 | 1.00 - 1.01 | 0.34 |
|  | 5 years | 1767 | 1.03 | 1.02 - 1.04 | < 0.01 |
|  | 10 years | 1132 | 1.05 | 1.03 - 1.07 | < 0.01 |
|  | 15 years | 732 | 1.05 | 1.02 - 1.08 | 0.01 |
|  | 20 years | 430 | 1.06 | 1.02 - 1.10 | < 0.01 |
| Papillary | 1 year | 2030 | 1.00 | 1.00–1.01 | 0.03 |
|  | 5 years | 1339 | 1.03 | 1.02–1.04 | < 0.01 |
|  | 10 years | 843 | 1.07 | 1.05–1.09 | < 0.01 |
|  | 15 years | 531 | 1.06 | 1.03–1.10 | < 0.01 |
|  | 20 years | 316 | 1.08 | 1.03–1.13 | < 0.01 |
| Follicular | 1 year | 624 | 1.00 | 0.98–1.01 | 0.57 |
|  | 5 years | 428 | 1.01 | 0.98–1.04 | 0.61 |
|  | 10 years | 289 | 1.00 | 0.95–1.05 | 0.96 |
|  | 15 years | 201 | 1.01 | 0.95–1.08 | 0.69 |
|  | 20 years | 114 | 1.00 | 0.91–1.08 | 0.91 |
| Female | 1 year | 1847 | 1.00 | 1.00 - 1.01 | 0.11 |
|  | 5 years | 1232 | 1.03 | 1.02 - 1.05 | < 0.01 |
|  | 10 years | 792 | 1.07 | 1.05 - 1.09 | < 0.01 |
|  | 15 years | 528 | 1.08 | 1.05 - 1.12 | < 0.01 |
|  | 20 years | 299 | 1.08 | 1.03 - 1.13 | < 0.01 |
| Male | 1 year | 807 | 1.00 | 0.99 - 1.01 | 0.69 |
|  | 5 years | 535 | 1.01 | 0.98 - 1.03 | 0.49 |
|  | 10 years | 340 | 1.01 | 0.97 - 1.05 | 0.58 |
|  | 15 years | 204 | 0.98 | 0.92 - 1.04 | 0.48 |
|  | 20 years | 131 | 1.02 | 0.94 - 1.09 | 0.67 |

Abbreviations: CI: confidence interval; DTC: differentiated thyroid cancer; RS: relative survival

**Supplement Table 2** UICC Stages in comparison to matched general population

| Relative Survival | | Number [n] | RS | 95% CI | P value |
| --- | --- | --- | --- | --- | --- |
| I | 1 year | 1988 | 1.01 | 1.01 – 1.01 | < 0.01 |
|  | 5 years | 1344 | 1.04 | 1.03 – 1.04 | < 0.01 |
|  | 10 years | 888 | 1.07 | 1.05 – 1.08 | < 0.01 |
|  | 15 years | 607 | 1.09 | 1.06 – 1.11 | < 0.01 |
|  | 20 years | 373 | 1.13 | 1.09 – 1.17 | < 0.01 |
| II | 1 year | 312 | 1.02 | 1.01 – 1.04 | < 0.01 |
|  | 5 years | 202 | 1.07 | 1.03 – 1.12 | < 0.01 |
|  | 10 years | 125 | 1.14 | 1.05 – 1.22 | < 0.01 |
|  | 15 years | 57 | 1.01 | 0.87 – 1.16 | 0.84 |
|  | 20 years | 25 | 0.95 | 0.74 – 1.16 | 0.67 |
| III | 1 year | 28 | 0.99 | 0.89 – 1.08 | 0.78 |
|  | 5 years | 19 | 1.15 | 1.01 – 1.29 | 0.03 |
|  | 10 years | 14 | 1.49 | 1.23 – 1.75 | < 0.01 |
|  | 15 years | 8 | 1.81 | 1.11 – 2.50 | 0.02 |
|  | 20 years | 5 | 2.46 | 1.01 – 3.91 | 0.05 |
| IVa | 1 year | 10 | 1.09 | 1.09 – 1.09 | < 0.01 |
|  | 5 years | 6 | 1.00 | 0.54 – 1.46 | 0.99 |
|  | 10 years | 3 | 1.30 | 0.35 – 2.26 | 0.53 |
|  | 15 years | 1 | 3.40 | 0.92 – 5.88 | 0.06 |
|  | 20 years | N/A | N/A | N/A | N/A |
| IVb | 1 year | 126 | 0.89 | 0.83 – 0.96 | < 0.01 |
|  | 5 years | 63 | 0.80 | 0.68 – 0.92 | < 0.01 |
|  | 10 years | 29 | 0.72 | 0.53 – 0.92 | < 0.01 |
|  | 15 years | 11 | 0.84 | 0.49 – 1.18 | 0.35 |
|  | 20 years | 3 | 0.65 | 0.03 – 1.27 | 0.28 |
| unknown | 1 year | 190 | 1.00 | 0.97 – 1.03 | 0.73 |
|  | 5 years | 133 | 1.04 | 0.98 – 1.10 | 0.19 |
|  | 10 years | 73 | 1.06 | 0.94 – 1.17 | 0.33 |
|  | 15 years | 48 | 1.01 | 0.85 – 1.18 | 0.90 |
|  | 20 years | 24 | 0.86 | 0.64 – 1.08 | 0.21 |

Abbreviations: CI: confidence interval; UICC: Union for International Cancer Control; RS: relative survival

**Supplement Table 3** age-groups in comparison to matched general population

| Relative Survival | | Number [n] | RS | 95% CI | P value |
| --- | --- | --- | --- | --- | --- |
| < 30 years | 1 year | 364 | 1.00 | 0.99 – 1.00 | 0.50 |
|  | 5 years | 247 | 0.99 | 0.97 – 1.00 | 0.09 |
|  | 10 years | 177 | 0.99 | 0.97 – 1.01 | 0.25 |
|  | 15 years | 134 | 0.99 | 0.97 – 1.17 | 0.50 |
|  | 20 years | 97 | 1.00 | 0.97 – 1.27 | 0.94 |
| 30 - < 40 years | 1 year | 462 | 1.00 | 1.00 – 1.00 | < 0.01 |
|  | 5 years | 324 | 1.01 | 1.00 – 1.01 | 0.15 |
|  | 10 years | 216 | 1.01 | 1.00 – 1.03 | 0.04 |
|  | 15 years | 146 | 1.02 | 1.00 – 1.05 | 0.05 |
|  | 20 years | 102 | 1.06 | 1.03 – 1.08 | < 0.01 |
| 40 - < 55 years | 1 year | 909 | 1.00 | 1.00 – 1.01 | 0.41 |
|  | 5 years | 610 | 1.01 | 0.99 – 1.02 | 0.40 |
|  | 10 years | 398 | 1.02 | 0.99 – 1.04 | 0.19 |
|  | 15 years | 283 | 1.03 | 0.99 – 1.07 | 0.11 |
|  | 20 years | 166 | 1.13 | 1.07 – 1.18 | < 0.01 |
| 55 < 70 years | 1 year | 676 | 1.01 | 1.00 – 1.02 | 0.12 |
|  | 5 years | 452 | 1.06 | 1.03 – 1.09 | < 0.01 |
|  | 10 years | 274 | 1.18 | 1.12 – 1.24 | < 0.01 |
|  | 15 years | 147 | 1.39 | 1.27 – 1.50 | < 0.01 |
|  | 20 years | 59 | 1.69 | 1.43 – 1.94 | < 0.01 |
| 70 - < 80 years | 1 year | 215 | 1.00 | 0.96 – 1.04 | 0.97 |
|  | 5 years | 122 | 1.28 | 1.17 – 1.38 | < 0.01 |
|  | 10 years | 61 | 1.96 | 1.65 – 2.26 | < 0.01 |
|  | 15 years | 22 | 3.45 | 2.44 – 4.46 | < 0.01 |
|  | 20 years | 6 | 4.27 | 1.17 – 7.37 | 0.04 |
| ≥ 80 years | 1 year | 28 | 1.02 | 0.86 – 1.18 | 0.79 |
|  | 5 years | 12 | 1.58 | 0.96 – 2.21 | 0.07 |
|  | 10 years | 6 | 5.32 | 1.86 – 8.79 | 0.01 |
|  | 15 years | – | – | – | – |
|  | 20 years | – | – | – | – |

Abbreviations: CI: confidence interval; RS: relative survival
